# Supplementary material for: A chromosome-level genome of Astyanax mexicanus surface fish for comparing population-specific genetic differences contributing to trait evolution
Source: Nat Commun. 2021 Mar 4;12:1447. doi: 10.1038/s41467-021-21733-z (PMC7933363; doi:10.1038/s41467-021-21733-z)
Supplement: Supplementary file 1 — Supplementary Information [file 41467_2021_21733_MOESM1_ESM.pdf]

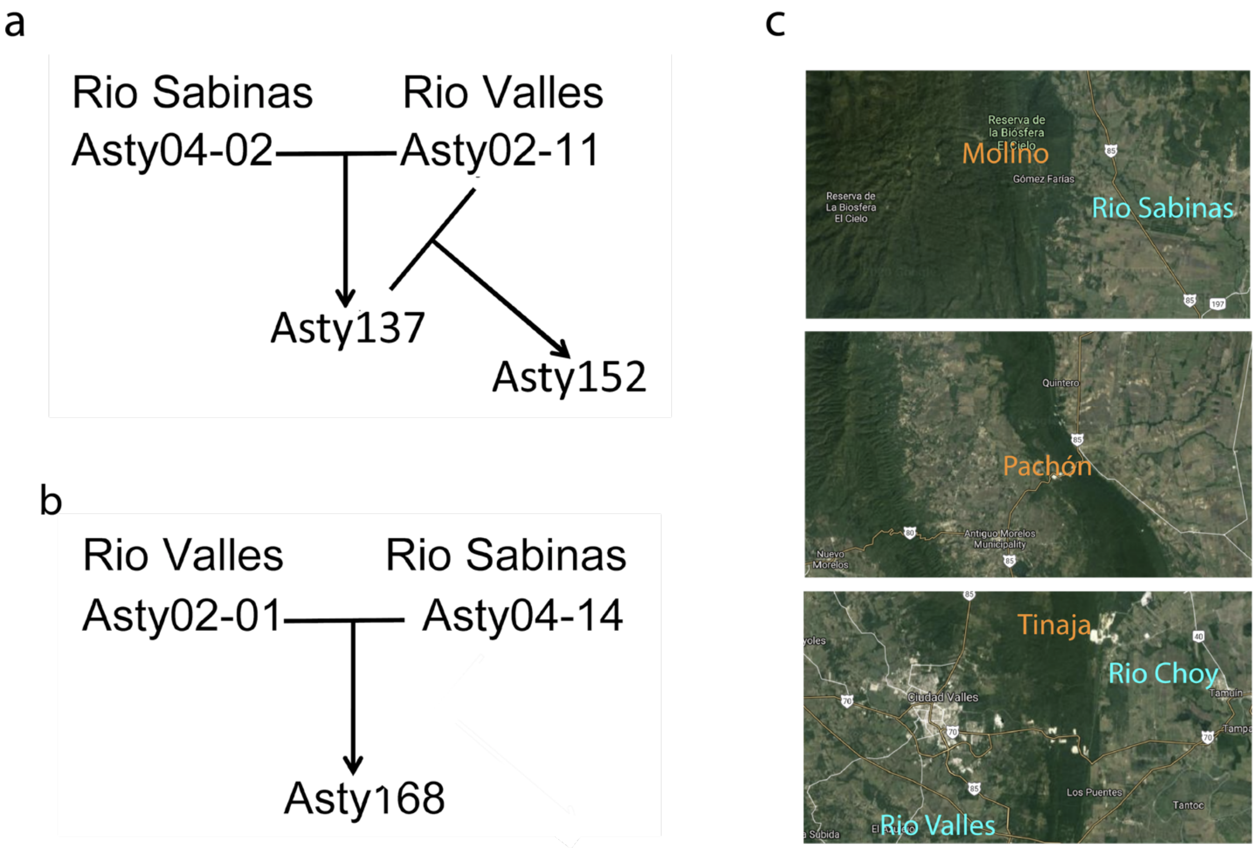

Supplementary Figure 1. Breeding scheme to cross two surface fish populations from different geographic locations in Mexico. (a) a female Río Sabinas (Asty04) was crossed to a male Río Valles (Asty02) and the resulting female (Asty 137) was crossed to the male Río Valles (Asty02). The resulting female fish (Asty152) was sequenced and assembled. (b) A female Río Valles (Asty02) was crossed to a male Río Sabinas (Asty04) and the resulting female was used for the BioNano Irys "restriction" map. c) locations of the rivers (blue) and caves (orange) discussed in the study. Maps from Google Maps.

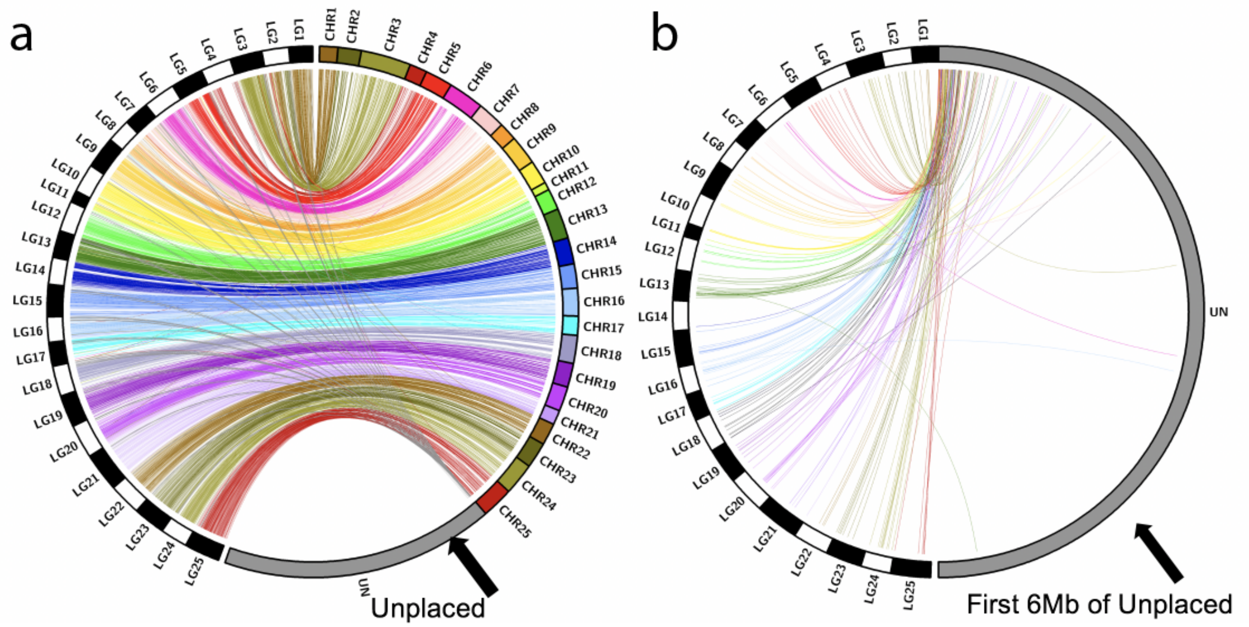

Supplementary Figure 2. Synteny between a recombination-based linkage map and the Astyanax mexicanus 2.0 reference. By mapping the relative positions of GBS markers from a dense map constructed from a Pachón x surface fish F_2 pedigree, we observed significant synteny based on 96.6% of our markers (a). Although a substantial portion of the genome remains unplaced (grey), we noted sparse distribution of GBS markers beyond the first 6 Mb of the unplaced scaffold (represented in grey, (b). The majority of these alignments were present in the first 1 Mb of the unplaced scaffold (grey). This syntenic representation indicates substantial structural similarity between the cavefish and surface genomes and demonstrates the utility of the draft surface fish genome for identifying QTL intervals and nominating candidate genes associated therein.

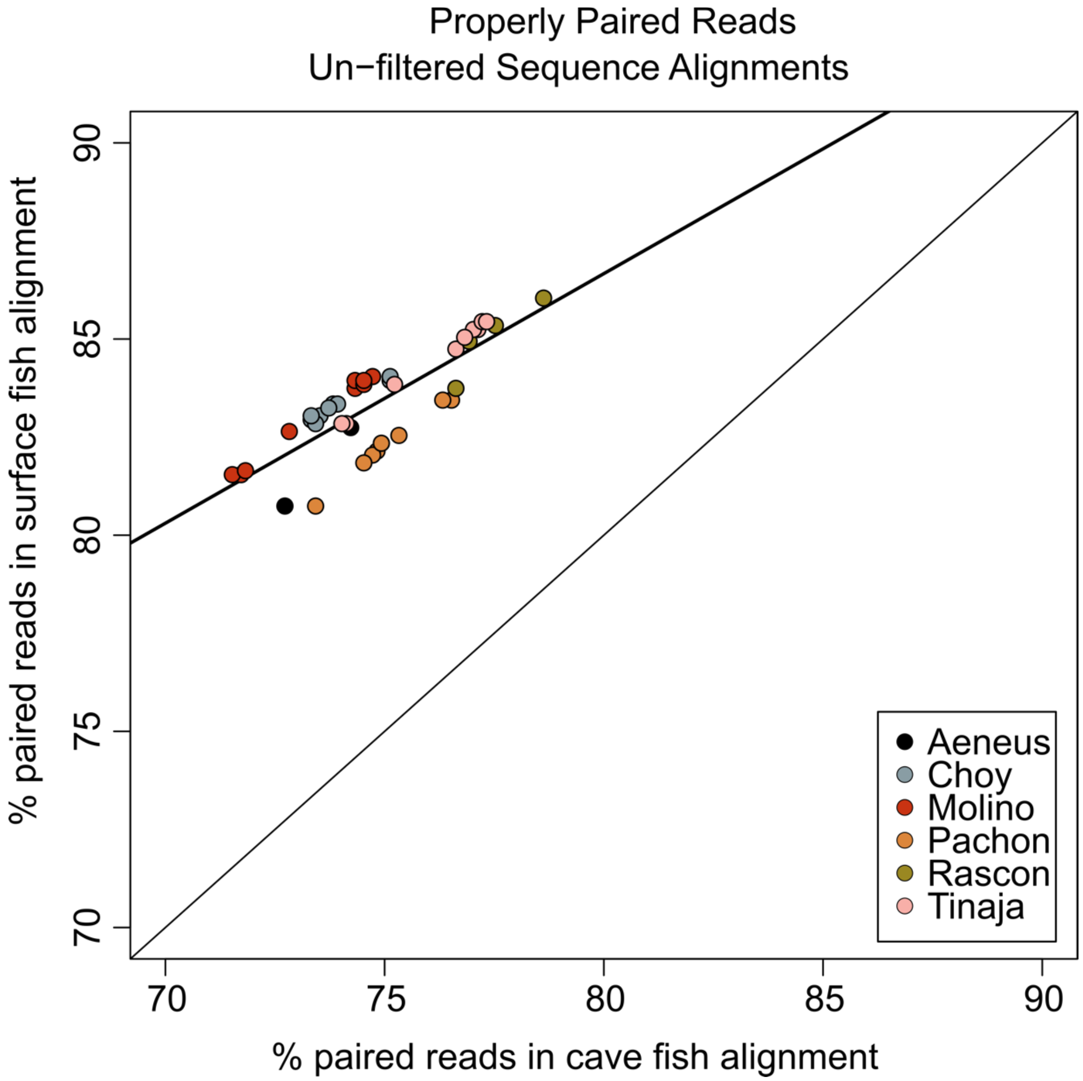

Supplementary Figure 3. Percentage of properly paired reads for each sample aligned to the surface (y-axis) and cave (x-axis) reference genomes. Samples are colored by population identity. Line is best fit of all samples. Properly paired reads include reads that align in the correct orientation to the same scaffold/chromosome in the assembly. The greater proportion of properly pairing reads in the alignment to the surface genome likely reflects the greater level of contiguity of the surface assembly, e.g, N50 contig length.

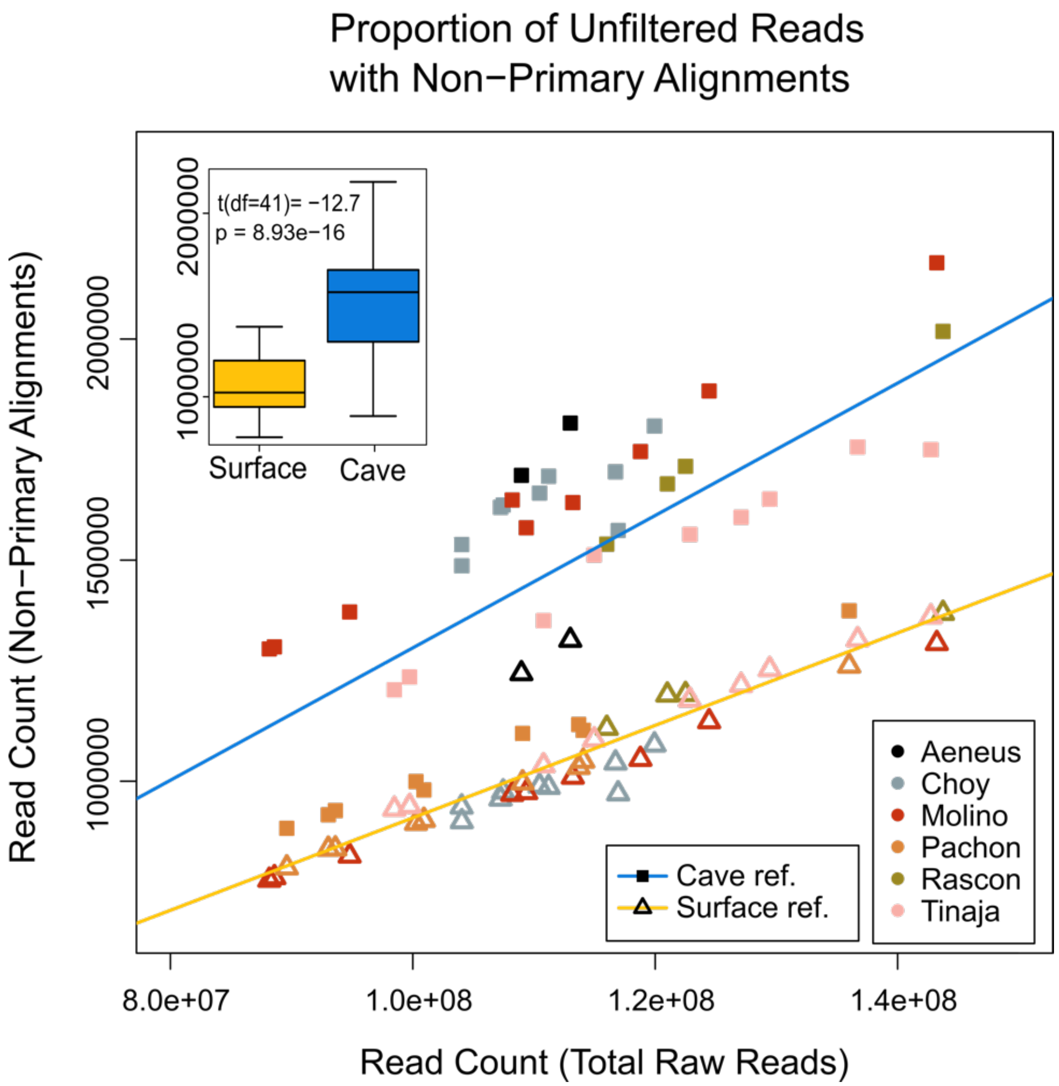

Supplementary Figure 4. Non-primary read alignment counts for samples aligned to the surface (y-axis) and cave (x-axis) reference genomes. The reference alignment for each sample is indicated by shape (cave reference: square, surface reference: triangle). Colored lines are fit to samples aligned to each respective genome (cave: blue, surface: yellow). Each sample (15 surface and 27 cave fish samples) was aligned to both reference genomes and are united by color indicating population membership. Inset boxplots are the lower and upper quartiles and median with minimum/maximum whiskers. The inset boxplot includes the results of a two-sided paired t-test (t=-12.7, df=41, p=8.93e-16, N = 42) between the number of non-primary alignments for each sample aligned to each genome. Non-primary alignments include reads that have multiple mapping positions and reads with chimeric alignments. Alignment to the cave results in significantly more non-primary alignments with greater variation among the aligned samples.

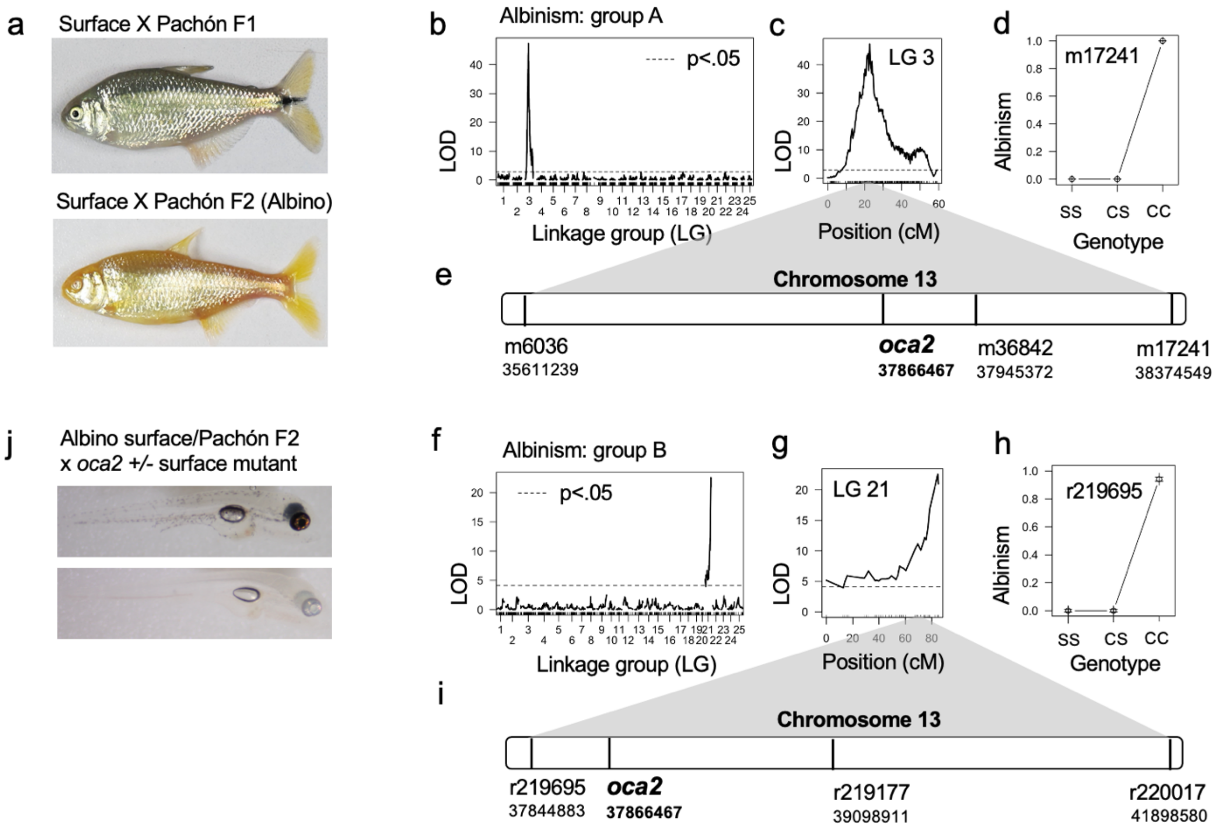

Supplementary Figure 5. Utilizing the surface fish genome for QTL mapping of albinism. a) Representative image of F_1 hybrid of surface/Pachón cross and albino F_2 hybrid. b-i) QTL mapping of albinism in two independent surface/Pachón QTL mapping studies: group A (b-e) and group B (f-i). b, f) Results of genome wide LOD calculation for albinism using Haley-Knott regression and binary model (0 = pigmented, 1 = albino). Significance threshold of 5% (black dotted line) determined by calculating the 95th percentile of genome-wide maximum penalized LOD score using 1000 random permutations. c, g) LOD score for each marker on the linkage group with the peak marker. d, h) Plot highlighting the effect of the indicated genotype at the peak marker (S = surface allele, C = cave allele). e, i) Although the albinism QTL map to separate linkage groups (LG3 and LG21) in two independent mapping studies, aligning the markers that define the 1.5 LOD support intervals to the new surface fish genome reveals the position of the QTL on chromosome 13 near the *oca2* gene. b) Pigmented (top) and albino (bottom) progeny resulting from breeding a surface/Pachón albino F_2 hybrid with a surface fish heterozygous for an engineered 4 base pair deletion in *oca2* exon 21.

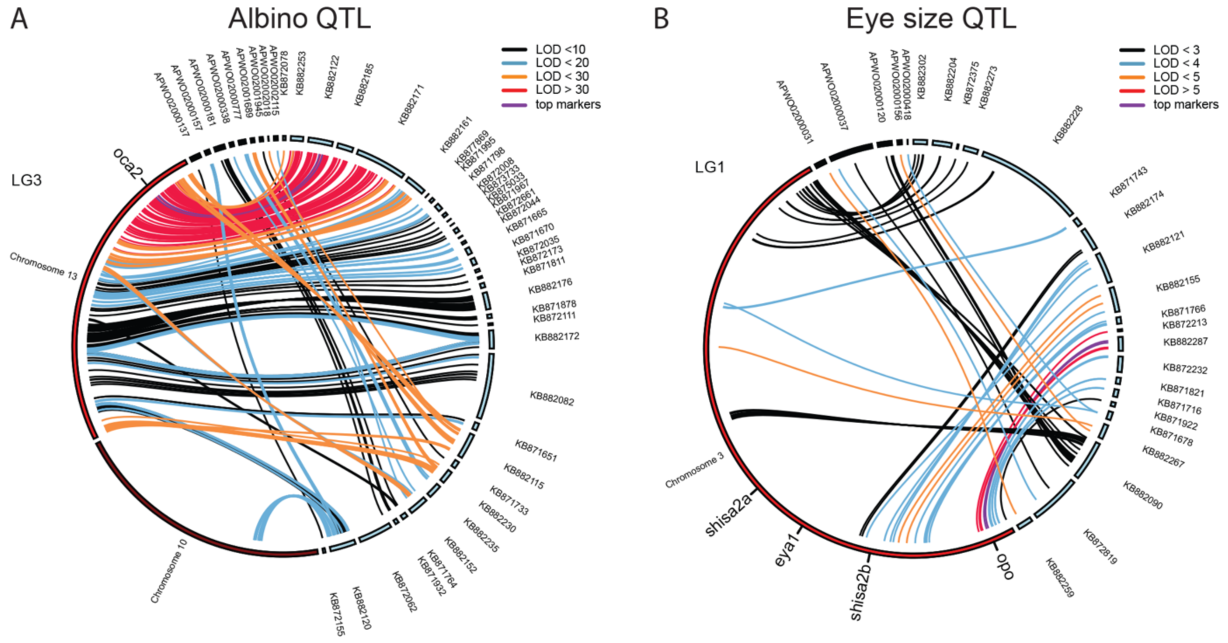

Supplementary Figure 6. Surface fish genome allows for long and continuous intervals in the QTL analysis. Circos plot for albino QTL (a) and eye size QTL (b) showing the markers with significant LOD scores on both the cavefish (blue contigs) and surface fish genome (red/black contigs).

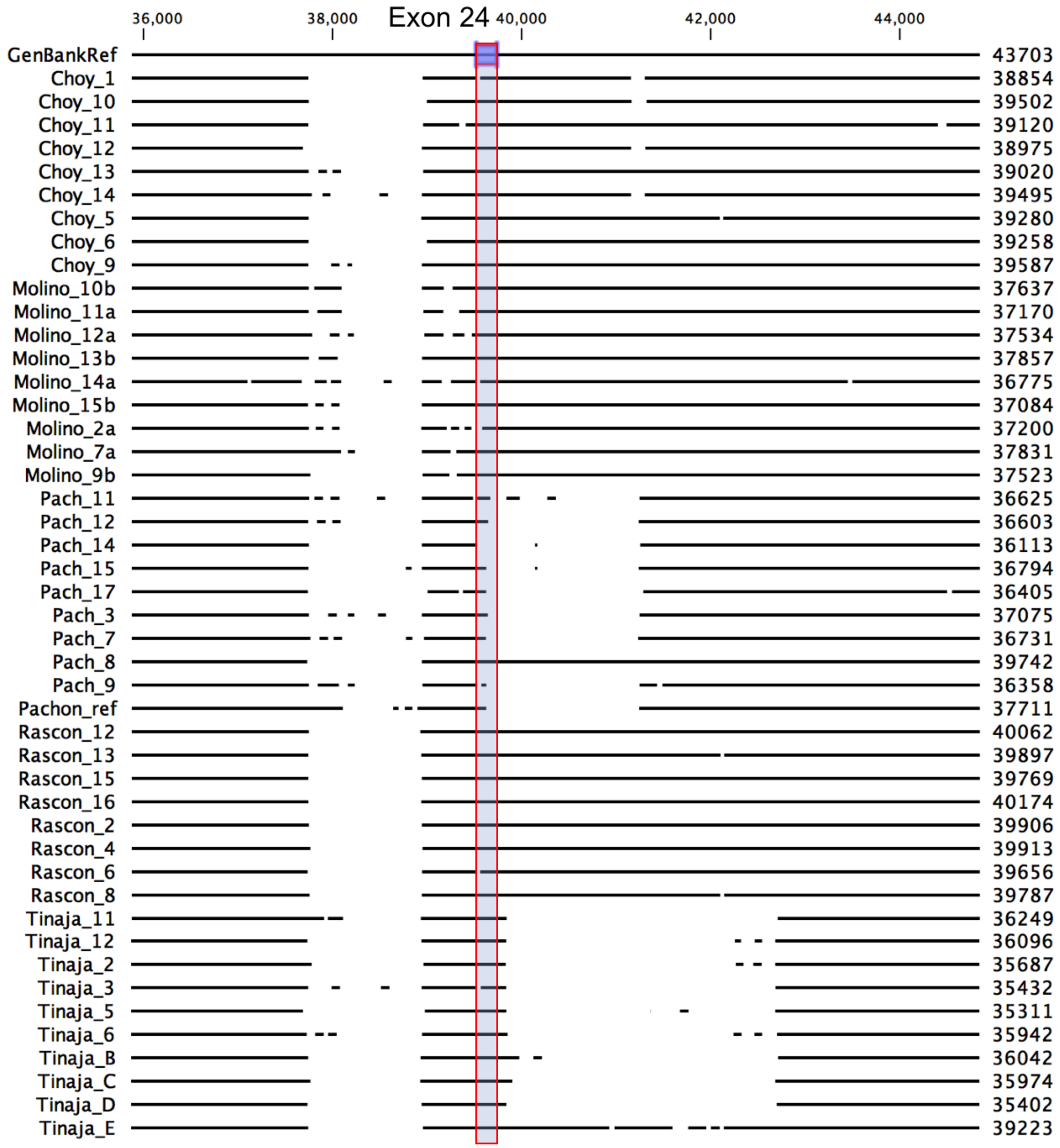

Supplementary Figure 7. Alignment of *oca2* exon 24 sequencing data from wild samples utilizing the Astyanax mexicanus 2.0 as a reference. Portion of oca2 locus on chromosome 13 of the annotated surface fish genome (top) aligned to sequencing data from wild-caugh surface fish (Río Choy, Rascón) and cavefish (Molino, Pachón, Tinaja). Exon 24 is highlighted and gaps in horizontal lines indicated sequence deletion. Alignments made using CLC sequence viewer.

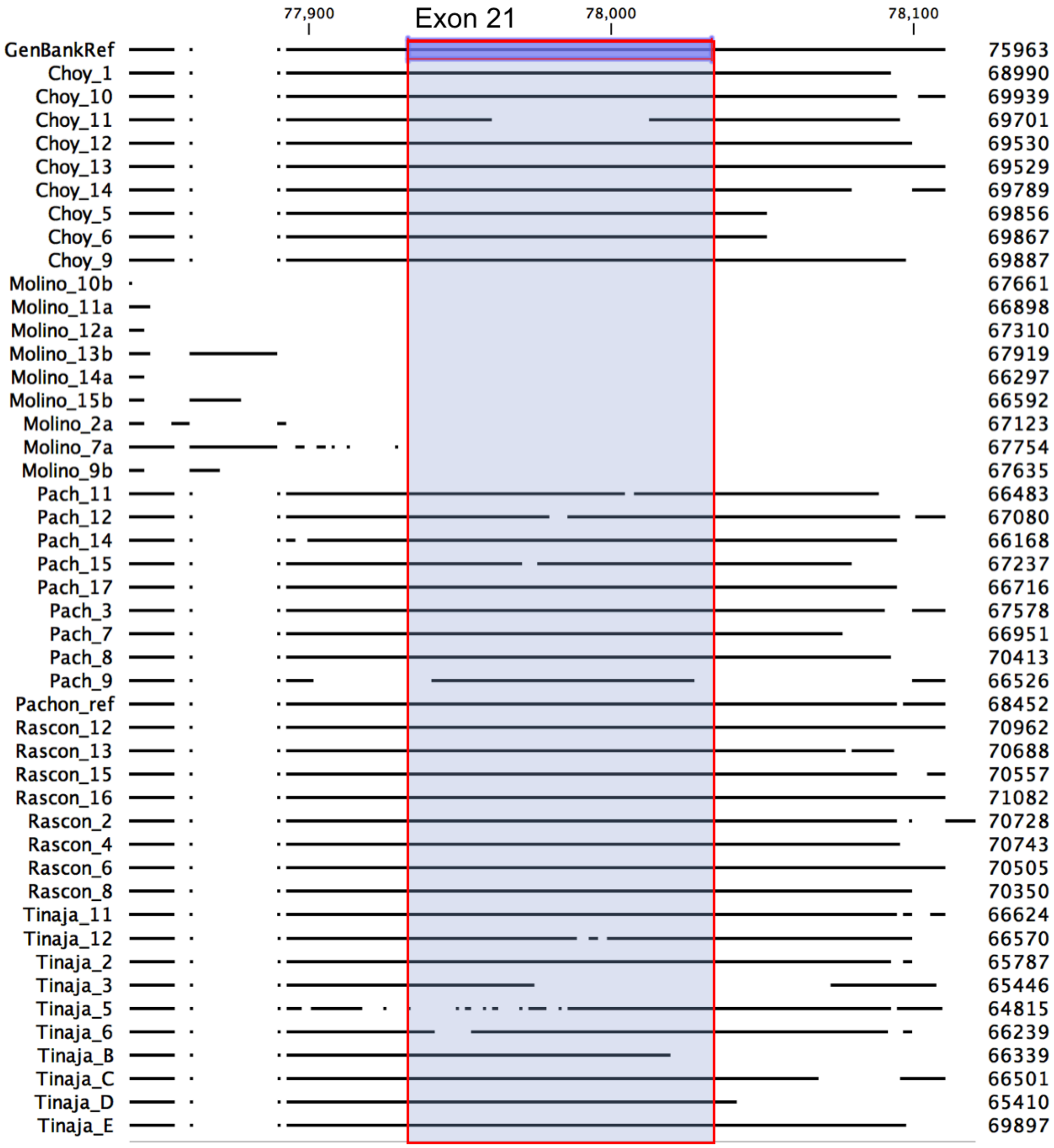

Supplementary Figure 8. Alignment of *oca2* exon 21 sequencing data from wild samples utilizing the Astyanax mexicanus 2.0 as a reference. Portion of *oca2* locus on chromosome 13 of the annotated surface fish genome (top) aligned to sequencing data from wild-caugh surface fish (Río Choy, Rascón) and cavefish (Molino, Pachón, Tinaja). Exon 24 is highlighted and gaps in horizontal lines indicated sequence deletion. Alignments made using CLC sequence viewer.

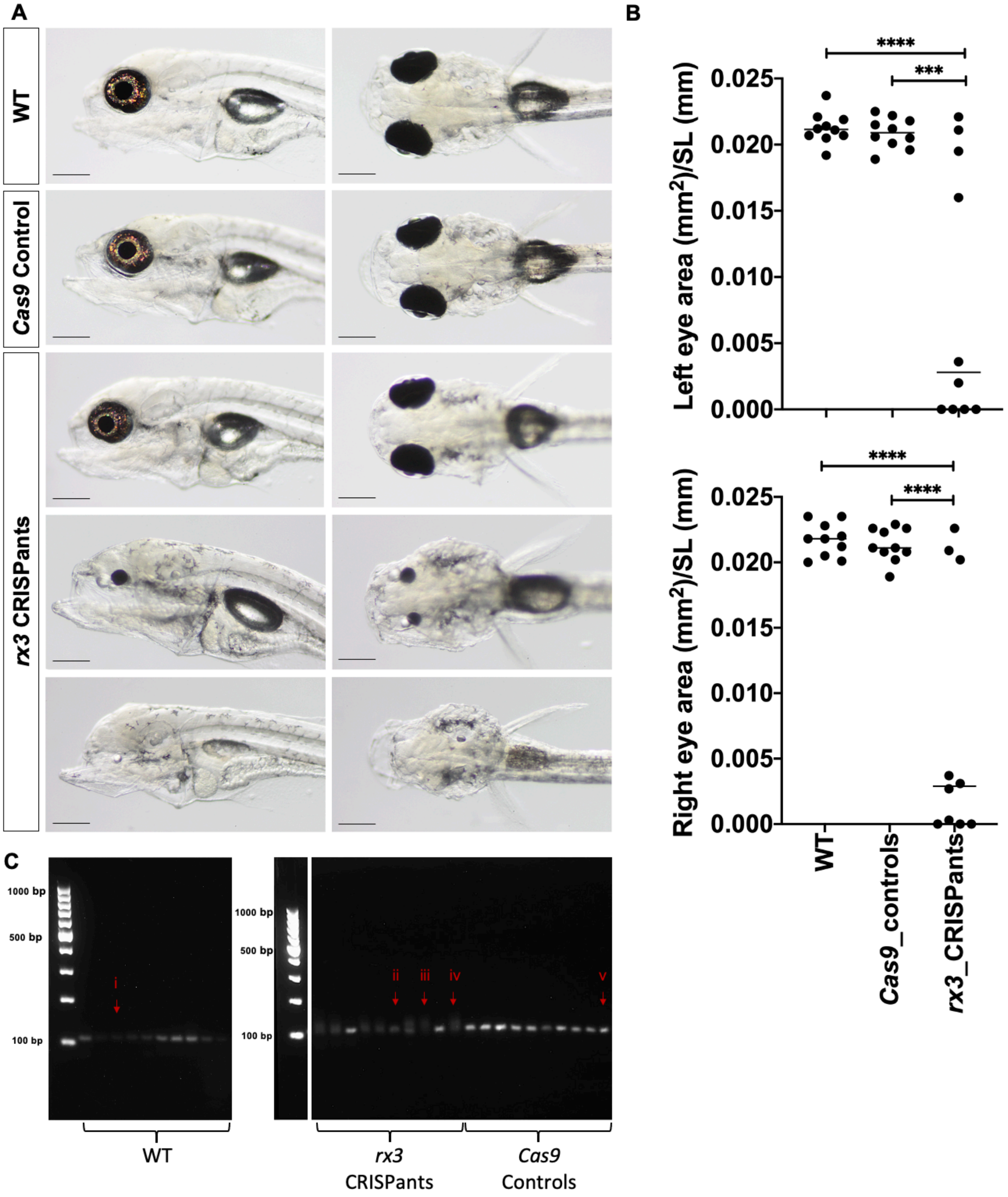

Supplementary Figure 9. Eye defects in larval *rx3* CRISPant fish. A) Lateral and dorsal images of wild type (WT) controls, *Cas9* mRNA injected controls and *rx3* CRISPant fish at 6 days post fertilization, showing the morphology of the eyes. Both, WT and *Cas9*-injected controls have large, well developed eyes, while *rx3* CRISPant fish have eyes that range in size. Scale bar = 250µm. B) Left and right eye area was measured in WT individuals, *Cas9* injected controls and *rx3* CRISPant fish (n=10 larvae each), showing the reduction in eye size in the *rx3* CRISPant fish compared with the two controls (Left eye; One-way ANOVA: F=15.97, $p<0.0001$, posthoc Tukey test: WT vs *rx3* CRISPant: $p<0.0001$, Cas9 controls vs rx3 CRISPant: p=0.0001. Right eye; One-

way ANOVA: F=20.67 , p<0.0001, posthoc Tukey test: WT vs *rx3* CRISPant: p<0.0001, Cas9 control vs *rx3* CRISPants: p<0.0001). Data are presented in a column scatter graph showing the distribution of every data point along with the median. C) PCR products from the region surrounding the *rx3* gRNA target site were examined for mutagenesis by gel electrophoresis. The WT, *Cas9*-injected and *rx3* CRISPant individuals imaged in A and used for the measurements and statistical analysis in B were genotyped by PCR and visualized on a 3% agarose gel. Both WT and Cas9 injected control bands were sharp, while *rx3* CRISPants showed different severities of mutagenesis. Mutagenesis is indicated by a 'smeary' band, suggesting the presence of multiple alleles of different sizes at the locus. The red arrows correspond to the fish imaged in panel A (i=WT; ii-iv=rx3 CRISPants with bigger eyes (ii), smaller eyes (iii) and no eyes (iv); and v=Cas9 control). Note that the three *rx3* CRISPant individuals in B with large eyes show no to low mutagenesis in C (individuals 3, 6 and 9 of the *rx3* CRISPant set), further suggesting this phenotype is due to presence of mutant alleles in *rx3* CRISpant fish. Data shown are representative results from a total of 1 experiment. Uncropped gels can be found as Supplementary Figures 14 and 15 respectively.

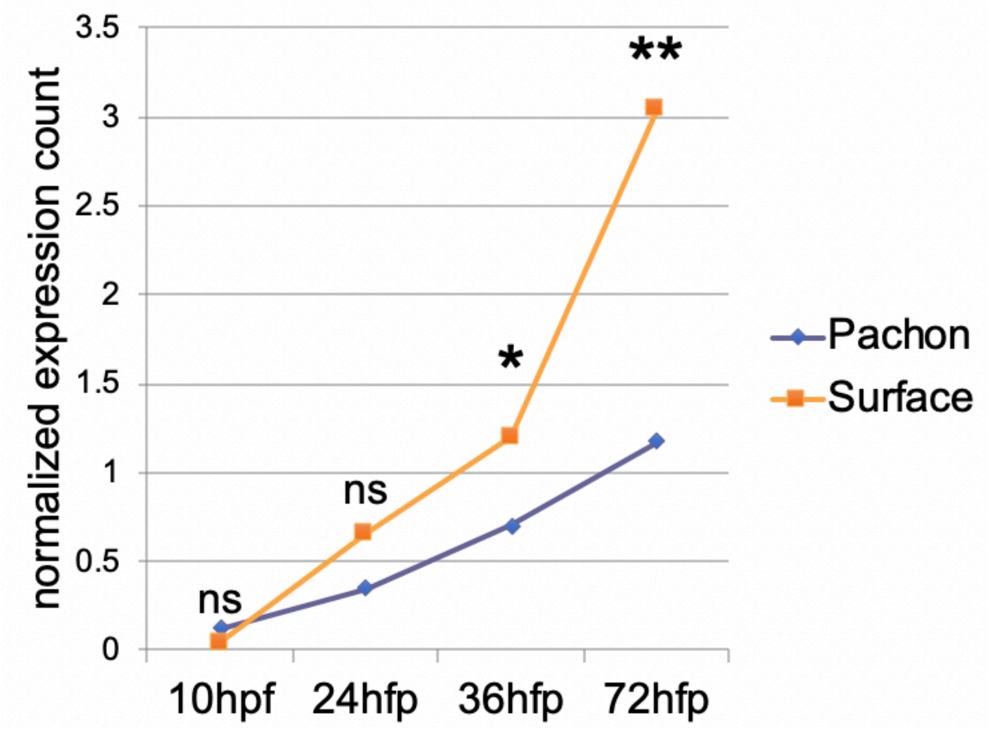

Supplementary Figure 10. Pachón cavefish have reduced expression of dusp26. Normalized expression count of dusp26 at the indicated stage of development. Significance codes; ns p>0.05, *p<0.05, **p<0.005 (p-values: 10hpf = 0.155; 24hpf = 0.243; 1.5dpf = 0.02; 3dpf = 0.003).

Transcriptomics data from: Stahl, B. A. & Gross, J. B. A. J Exp Zool B Mol Dev Evol 328, 515-532 (2017).

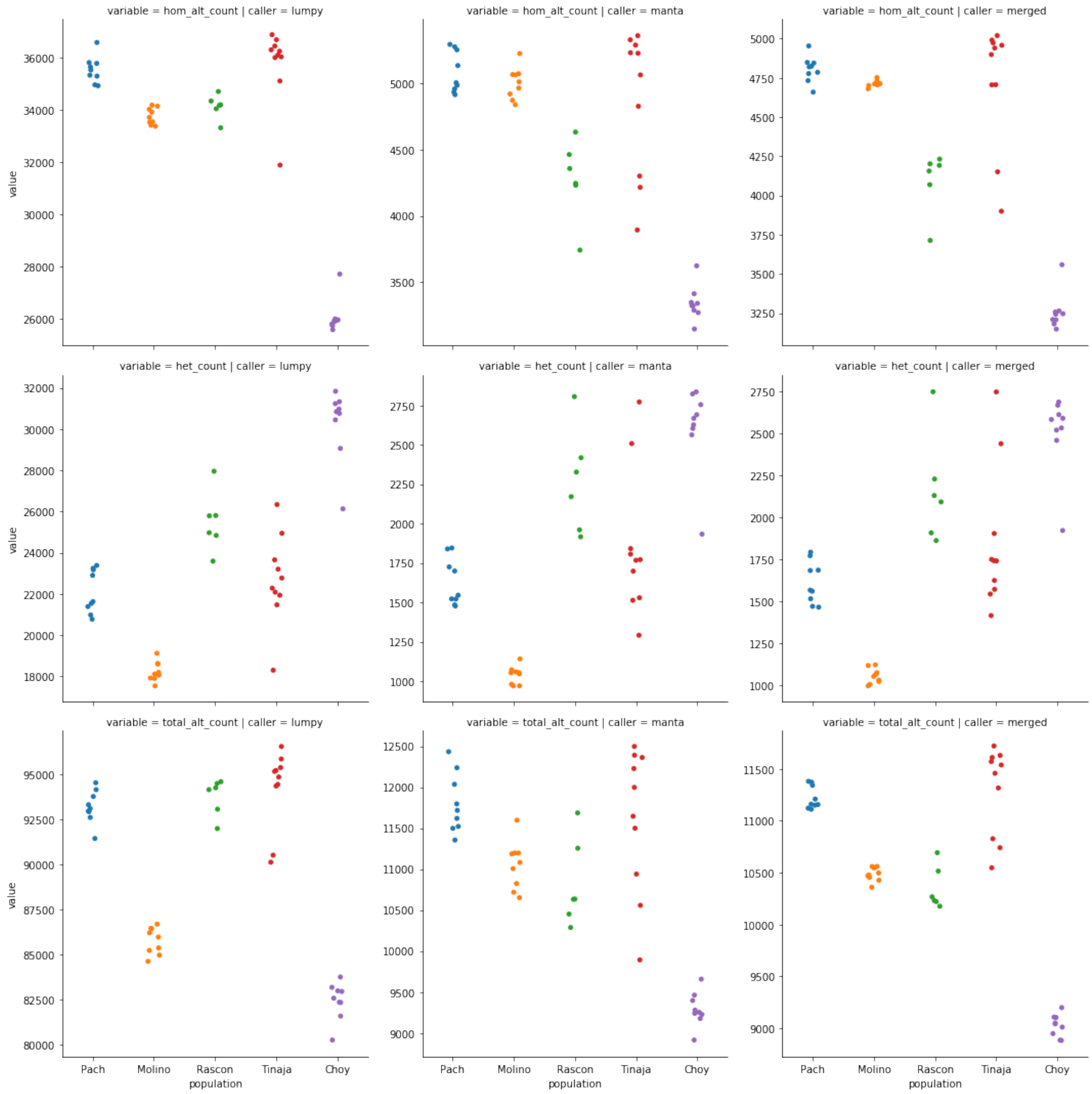

Supplementary Figure 11. Comparative counts of deletions called per sample and grouped by the samples' populations according to the SV caller. These deletions are parsed by homozygous or heterozygous state, or total number of haplotypes affected (rows), and by deletions detected made by lumpy, manta, or the intersection of the two sets (columns).

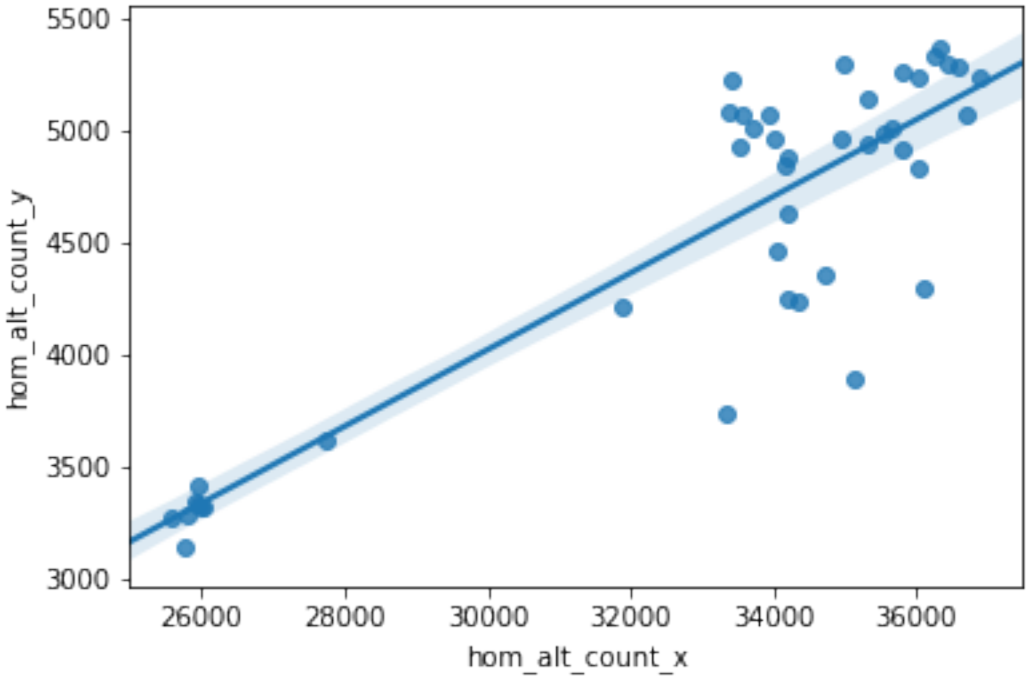

Supplementary Figure 12. Comparison of number of deletions called per sample for lumpy to manta SV detection algorithms. A high correlation between the results of the two callers is observed. The line shown is a linear regression model with the 95% confidence interval around it shaded.

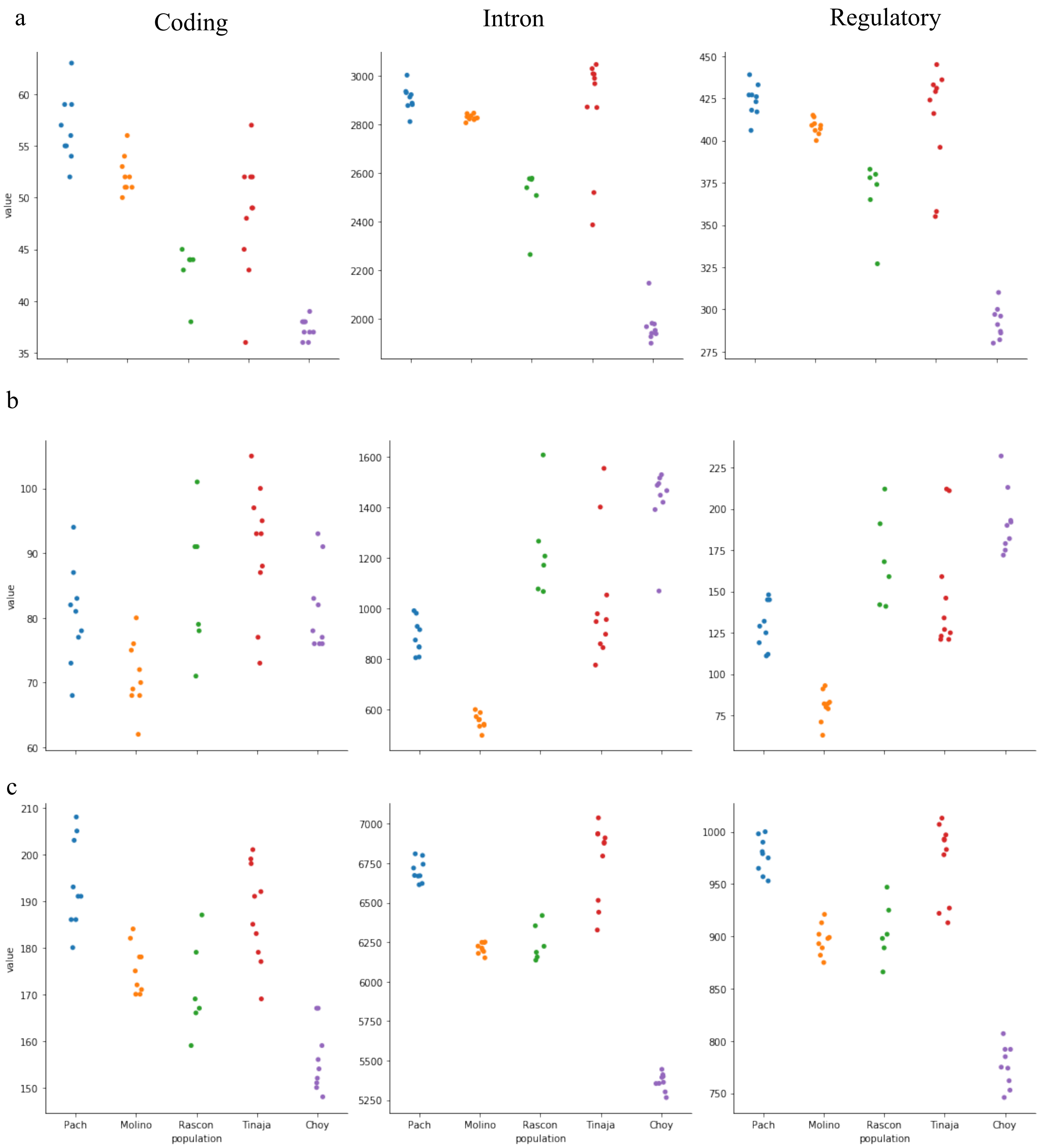

Supplementary Figure 13. Counts of deletions called per sample, grouped by the samples' population source. (a) Homozygous alternate, (b) heterozygous, and (c) total alternate deletions compared to the Astyanax mexicanus 2.0 reference. Values on the y axis represent total deletion counts. Across all allele types deletions affecting coding, intronic, or regulatory sequence are labelled

accordingly. Putative regulatory sequence was arbitrarily defined as 1 kb upstream of the start and stop codon.

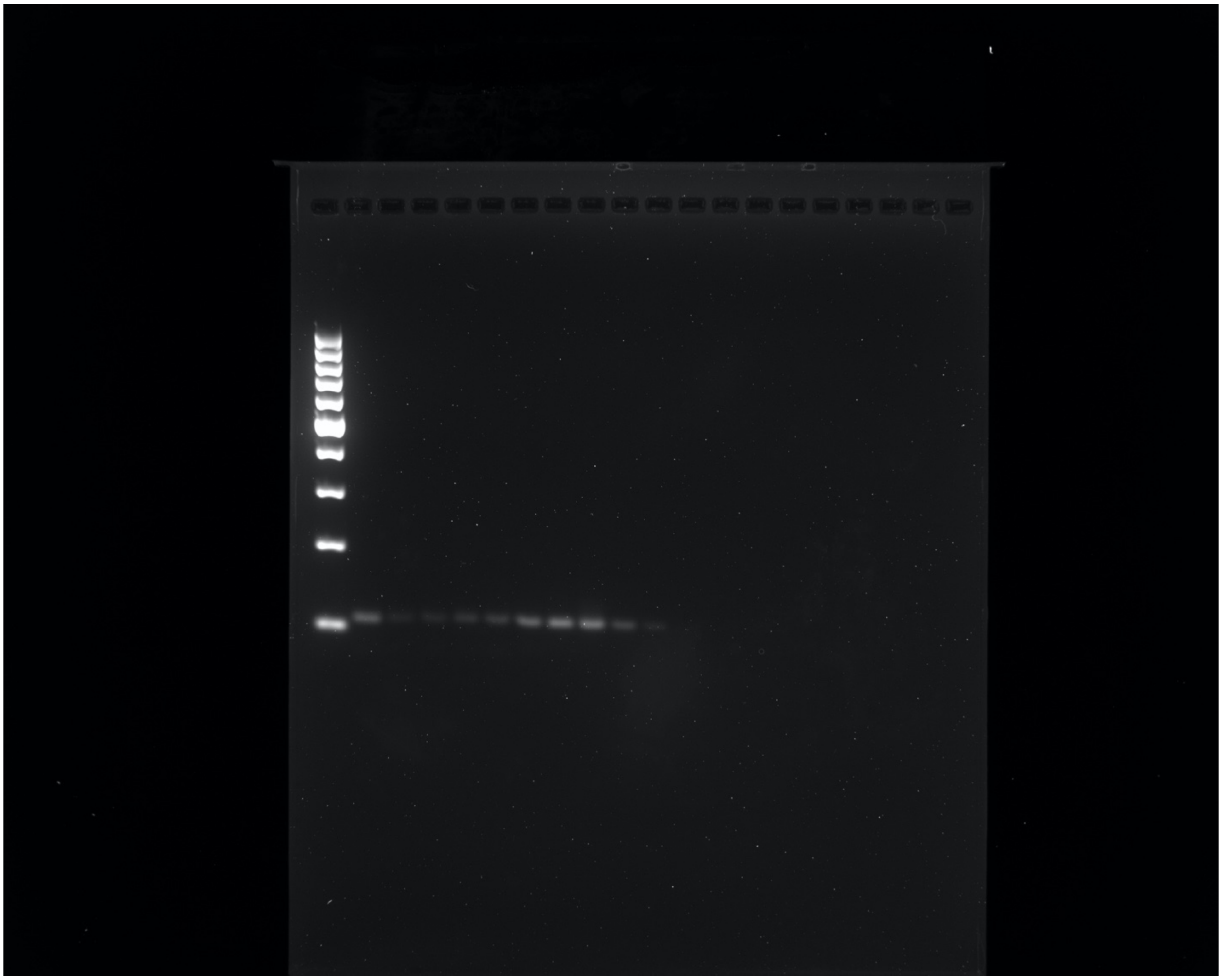

Supplementary Figure 14. Uncropped gel picture from supplementary Figure 9c.

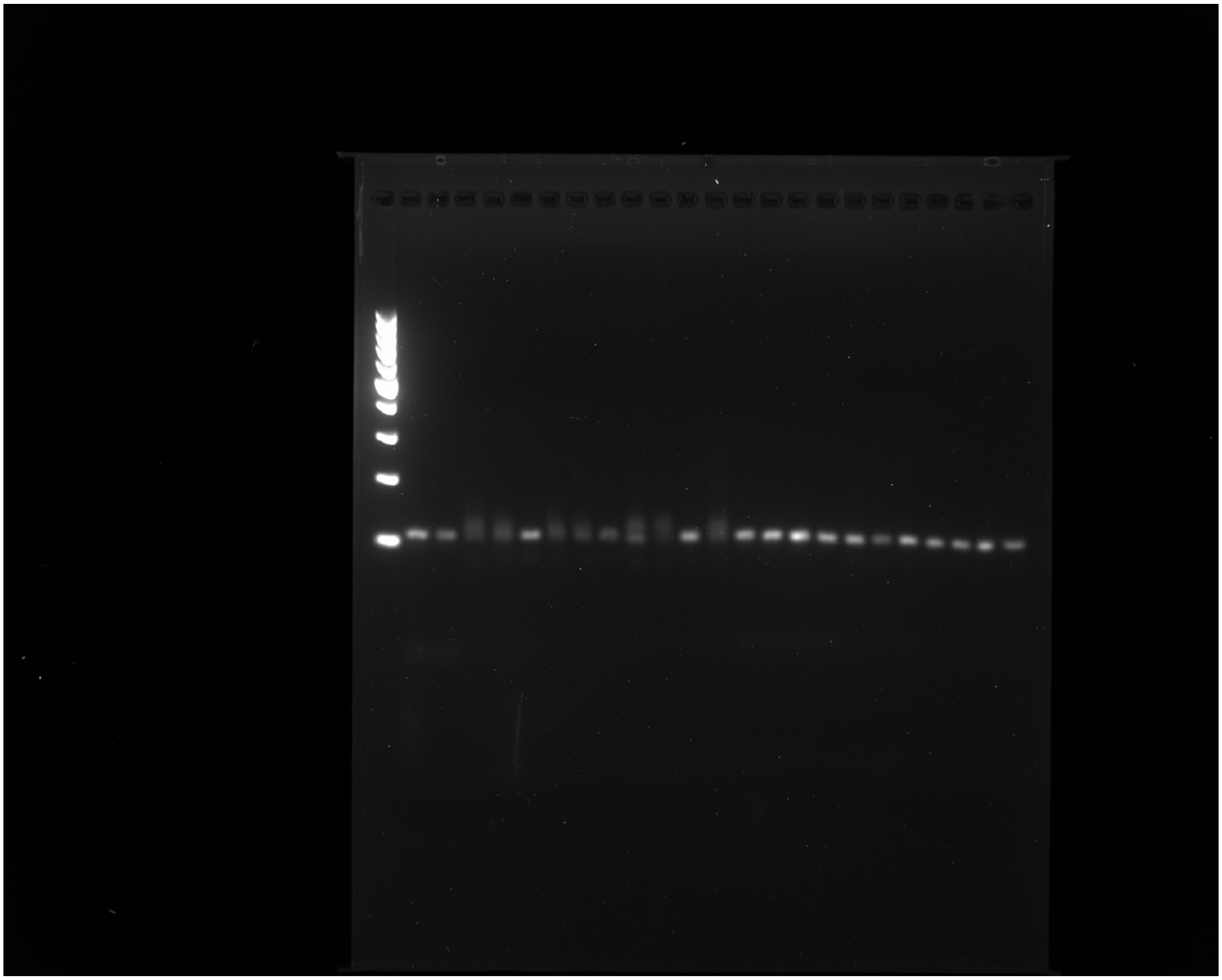

Supplementary Figure 15. Uncropped gel picture from supplementary Figure 9c.

Supplementary Table 1. Representative assembly metrics for sequenced teleost genomes[1].

Species	Assembled version	N50 contig (Mb)	N50 scaffold (Mb)	Total assembly size (Gb)	Total contigs	% Repeats[2]
Astyanax mexicanus (surface)	Astyanax mexicanus 2.0	1.76	35	1.29	3,030	41
Astyanax mexicanus (cave)	Astyanax mexicanus 1.0.2	0.014	1.77	1.19	121,345	30
Xiphophorus	X maculatus 5.0	9.1	31	0.7	258	27

maculatus	male					
Oryzias latipes	ASM223467v1	2.5	31	.73	516	34
Danio rerio	GRCz11	1.4	7.3	1.36	19,725	48

[1]All species-specific assembly metrics derived from the NCBI assembly archive.
[2]Total repeats estimated by WindowMasker

Supplementary Table 2. Representative gene annotation measures for assembled teleost genomes[1].

Species name	Assembled version	Protein coding genes	Total ncRNA	mRNAs
Astyanax mexicanus	Astyanax mexicanus 2.0	25,293; 26,698[2]	5,314	42,649
Astyanax mexicanus	Astyanax mexicanus 1.0.2	23,628	1,062	33,353
Xiphophorus maculatus	Xiphosphorus 5.0 male	23,238	3,620	43,551
Oryzias latipes	ASM223467v1	22,071	4,481	44,753
Danio rerio	GRCz11	26,522	13,137	52,818

[1]All species-specific gene annotation metrics derived from the NCBI RefSeq comparisons.
[2]Gene annotation metrics from Ensembl 98.

Supplementary Table 3. A summary of gene representation using highly conserved gene orthologs from multiple species.

Gene metric	%
Complete	94.6
Complete and single copy	90.4
Complete and duplicated	4.2
Fragmented	1.4
Missing	4.0

BUSCO was run in genome mode. A total of 4,584 conserved mammalian orthologs were used.

Supplementary Table 4. QTL mapping of albinism and eye size in two groups of surface/Pachón F_2 hybrids. Table comparing QTL marker positions on linkage maps, Pachón genome scaffolds, and surface fish genome chromosomes ([1] Astyanax mexicanus 1.0.2; [2] Astyanax mexicanus 2.0).

F_2 Surface/ Pachón	Phenotype	Marker	LOD	Linkage group position (cM)	Pachón scaffold position[1]	Surface chromosome position[2]
group A	albinism	m6036	45.03	3:22.6	KB882185: 1429320	13: 35611239
group A	albinism	m17241	47.31	3:22.6	KB882122: 1818424	13: 38374549
group A	albinism	m36842	45.16	3:22.8	KB882122: 2246330	13: 37945372
group B	albinism	r219177	20.67	22:22.5	KB882122: 1091706	13: 39098911
group B	albinism	r219695	22.56	22:24.6	KB882122: 2349092	13:37844883
group B	albinism	r220017	20.98	22:25.0	KB882122: 3270816	13: 41898580
group A	eye size	m23384	3.39	1:62.6	KB882121: 3061587	3: 23014157
group A	eye size	m15225	5.46	1:66.8	KB882287: 356685	3: 9301997
group A	eye size	m30438	3.89	1:67.9	KB872232: 7153	3: 8742127
group B	eye size	r230947	8.45	15:31.0	KB882132: 1682036	20: 1802669
group B	eye size	r231141	11.98	15:35.9	KB882132: 2321700	20: 1169105

Supplementary Table 5. Candidate genes in surface fish genome intervals underlying previously reported activity QTL in *Astyanax mexicanus*[1,2]. Candidate genes previously identified based on the Pachón cave genome (Astyanax mexicanus 1.0.2) are indicated with asterisks.

Ensembl Gene ID	Gene Name	Chromosomal Location
ENSAMXG00000021023	*smarca4a*	2:11818816-11837849:-1
ENSAMXG00000008438	*coro1a*	2:14117859-14135882:-1
ENSAMXG00000034332	*arl6ip1*	2:14385087-14398765:-1
ENSAMXG00000009999	*unc119.2*	2:14893606-14905509:-1
ENSAMXG00000011025	*kcnc3a*	2:15763673-15882326:-1
ENSAMXG00000002938	*alcamb*	2:19282937-19312685:-1
ENSAMXG00000003038	*lim2.3*	2:19536533-19540803:-1
ENSAMXG00000009792	*cryaba*	2:20946260-20947554:1
ENSAMXG00000032727	*tbx2b*	2:24487301-24496709:1
ENSAMXG00000010190	*mfrp*	2:25661299-25673051:1
ENSAMXG00000013648	*flr*	2:27598963-27629303:-1
ENSAMXG00000004873	*rpgrip1*	3:8845958-8869048:1
ENSAMXG00000029409	*tfap2a**	3:9109394-9133706:1

ENSAMXG00000026077	novel gene	3:12526447-12529953:-1
ENSAMXG00000029859	novel gene	3:12998126-13002944:1
ENSAMXG00000001701	novel gene	3:13006893-13010808:1
ENSAMXG00000001577	*crx*	3:13160896-13167893:-1
ENSAMXG00000006915	*npas2*	3:14232532-14258948:1
ENSAMXG00000038165	*tbx5b*	3:14702695-14713758:1
ENSAMXG00000016913	*tmtopsb**	3:17563872-17631603:-1
ENSAMXG00000014033	*chodl*	3:21723943-21733151:-1
ENSAMXG00000014704	*boc*	3:22384147-22424523:1
ENSAMXG00000014873	*klhl40b*	3:23009101-23016128:-1
ENSAMXG00000024536	*nek2*	3:31499309-31505232:-1
ENSAMXG00000024534	*ints7*	3:31581594-31598913:-1
ENSAMXG00000024533	*dtl*	3:31598791-31609823:1
ENSAMXG00000034080	*slc35b2*	3:31767337-31782355:1
ENSAMXG00000018645	*extl3*	3:32853820-32889965:1
ENSAMXG00000037688	*insm1a*	3:33509031-33510524:1
ENSAMXG00000043510	*zc4h2*	3:35812278-35820217:-1
ENSAMXG00000041759	*efnb1*	3:37303370-37355591:-1
ENSAMXG00000036821	*rhogc*	3:37574414-37575393:-1
ENSAMXG00000034538	*marcksa**	3:38448073-38453896:-1
ENSAMXG00000014261	*lama4*	3:38535994-38575406:1
ENSAMXG00000004758	*id2b*	3:41735650-41737485:-1
ENSAMXG00000001703	*sox11b*	3:42060037-42061293:-1
ENSAMXG00000002111	*nhsl1b*	3:42555719-42718544:1
ENSAMXG00000009797	*mcm3**	3:44339476-44347266:1
ENSAMXG00000030048	*opn8a*	3:44673077-44694625:1
ENSAMXG00000032634	*opn8b*	3:44710459-44727248:1
ENSAMXG00000034361	*napbb*	3:45437838-45448049:-1
ENSAMXG00000037668	*cipcb*	3:46387024-46395340:-1
ENSAMXG00000017253	*vps18*	3:56760089-56765547:1
ENSAMXG00000042064	*prph2lb*	3:57795222-57801758:1
ENSAMXG00000006177	*gmds*	3:58321161-58384972:1
ENSAMXG00000033985	*znf513a*	3:59487869-59503723:1
ENSAMXG00000019342	*hif1ab*	3:60162341-60177903:-1
ENSAMXG00000034914	*six6b*	3:60305080-60310494:-1
ENSAMXG00000020545	*jag2b*	3:61294730-61345304:-1
ENSAMXG00000040803	*opn7a*	3:62978088-63008081:-1
ENSAMXG00000005565	*clocka*	3:64430234-64454663:-1
ENSAMXG00000031528	*nmu*	3:64506864-64526902:-1
ENSAMXG00000005419	*paics*	3:64711940-64736902:1

ENSAMXG00000005200	*ipo13*	3:64958602-65019480:-1
ENSAMXG00000024961	*htr1b*	3:66681640-66682803:1
ENSAMXG00000010672	novel gene	3:66801298-66858990:1
ENSAMXG00000010179	*opn5**	3:73215451-73247349:1
ENSAMXG00000021415	*igf1rb**	5:27225260-27305403:1
ENSAMXG00000025528	*nfil3-5*	13:1256220-1257995:-1
ENSAMXG00000009984	*ttc26*	13:1431334-1441483:1
ENSAMXG00000038316	*tmtops2a*	13:7136778-7155237:1
ENSAMXG00000039940	*vapb**	13:9377160-9388944:1
ENSAMXG00000012711	*dmbx1b**	13:10553760-10563244:-1
ENSAMXG00000031725	*rtn4a*	13:12814377-12831725:-1
ENSAMXG00000016819	*scinla**	13:13069655-13089641:-1
ENSAMXG00000025156	*znf644b*	13:13495670-13547264:1
ENSAMXG00000004665	*sap130a*	13:16514965-16534778:1
ENSAMXG00000004743	*bcl6aa*	13:16670930-16676300:-1
ENSAMXG00000026091	*cdk5r2a*	13:17958856-17959791:1
ENSAMXG00000002203	*cryba2a*	13:17990940-18002011:-1
ENSAMXG00000002143	*desmb*	13:18080034-18091983:-1
ENSAMXG00000032420	*etv5b*	13:18190749-18209077:-1
ENSAMXG00000033167	*atf4a*	13:19522642-19530777:1
ENSAMXG00000011446	*pde6ha*	13:19633358-19633738:-1
ENSAMXG00000041726	novel gene	13:25481695-25483109:-1
ENSAMXG00000041979	*gli1*	13:28652595-28682666:-1
ENSAMXG00000039549	*tardbp*	13:29280168-29284278:-1
ENSAMXG00000024820	*kcna2a*	13:31980151-31981632:1
ENSAMXG00000016290	*setd5*	13:32341007-32354272:1
ENSAMXG00000033707	*mtmr14*	13:32429747-32448915:1
ENSAMXG00000030172	*emc3*	13:34518688-34525279:1
ENSAMXG00000002173	*ghrl*	13:36056678-36058841:-1
ENSAMXG00000035630	*cnga3a*	13:37221041-37236704:1
ENSAMXG00000012578	*rp2*	13:38348210-38355069:1
ENSAMXG00000035622	*rgs4**	13:40360036-40363859:-1
ENSAMXG00000038790	*b9d2**	14:1495190-1503124:1
ENSAMXG00000038525	*il7r*	14:2287140-2299347:-1
ENSAMXG00000005363	*rb1**	14:2978436-3034567:-1
ENSAMXG00000036655	*dlat**	14:3037422-3053639:-1
ENSAMXG00000040141	*rhogb*	14:6287379-6292949:-1
ENSAMXG00000037597	*nrxn2a*	14:8212904-8413941:1
ENSAMXG00000035067	*cryba1b*	14:8711160-8716854:-1

ENSAMXG00000024390	*unc119b*	14:8721262-8757794:1
ENSAMXG00000005573	*otpa*	14:12375640-12383285:1
ENSAMXG00000030762	*htr2cl2**	14:14798655-14808988:-1
ENSAMXG00000035790	*lhx5*	14:25157877-25177456:1
ENSAMXG00000014101	*gnrh2*	14:25303792-25305300:-1
ENSAMXG00000014133	*ptpra*	14:25310484-25364297:-1
ENSAMXG00000010561	*whrna*	14:30208193-30463006:-1
ENSAMXG00000037380	*rx3*	14:33825623-33831203:-1
ENSAMXG00000006642	*si:dkey-6b12.5*	14:35755277-35788288:-1
ENSAMXG00000016359	*sh3pxd2b*	14:36399980-36458373:-1
ENSAMXG00000002528	*kif3b*	15:21886044-21894243:-1
ENSAMXG00000043242	*dnmt3bb.2*	15:22917681-22935298:-1
ENSAMXG00000012860	*tnnc2*	15:25799535-25801853:1
ENSAMXG00000031060	*ca6*	15:27697722-27715234:-1
ENSAMXG00000025967	*atoh7**	25:14410275-14410697:1
ENSAMXG00000004732	*bag3*	25:14442551-14452067:1
ENSAMXG00000034070	*fgf8**	25:16916654-16924329:1
ENSAMXG00000012130	*chata**	25:17712720-17723271:-1
ENSAMXG00000042717	*rgra**	25:17736278-17746726:-1
ENSAMXG00000037212	*cxcl12a*	25:18837257-18847700:1
ENSAMXG00000002308	*zdhhc16a*	25:19360022-19366120:-1
ENSAMXG00000035696	*sfrp5**	25:19678068-19709202:-1
ENSAMXG00000029072	*pitx3**	25:21715018-21725509:1
ENSAMXG00000001987	*ninl*	25:21769890-21801028:-1

[1]See Carlson et al. (2018) for description of scored phenotypes and identified QTL intervals.

[2]Initial candidacy decision based on available Ensembl GO annotations only.

Supplementary Table 6. List of genes in the 1.5-LOD support interval for the eye size QTL in surface/Pachón F_2 hybrid group B (chromosome 20:1168905-1802909).

Gene name	Description
zswim6	zinc-finger SWIM-type containing
kif2a	kinesin family member 2a
kmt5aa	lysine methyltransferase 5aa
snmp35	small nuclear ribonucleoprotein 35 (U11/U12)
dusp26	dual specificity phosphatase 26
denr	density-regulated protein
hcar1	hydroxycarboxylic acid receptor 1
pde4d	phosphodiesterase 4D
depdc1b	DEP domain containing 1B

elovl7b	ELOVL fatty acid elongase 7b
pitpnm2	phosphatidylinositol transfer protein membrane associated 2
hip1r	huntingtin interacting protein 1 related
mphosph9	M-phase phosphoprotein 9
ccdc62	coiled-coil domain containing 62
cdk2ap1	cyclin-dependent kinase 2-associated protein 1-like
sbno	strawberry notch homolog 1 (Drosophila)
zmiz2	zinc finger, MIZ-type containing 2
ppiab	peptidylprolyl isomerase Ab (cyclophilin A)
rilpl1	Rab interacting lysosomal protein like 1
ENSAMXT00000043836	na
ENSAMXG00000040376	na
ENSAMXG00000038021	na

Supplementary Table 7. Astyanax mexicanus protein-coding genes with detected deletions across all cave populations (see Methods). Entrez gene symbols derived from NCBI gene annotation are provided.

ADGRG2	HLTF	LOC103025035
AP3B1	HNF4A	LOC103025085
ARL13A	HTT	LOC103025117
ASB5	IKZF1	LOC103025288
ASTN1	IZUMO1	LOC103025292
BAG3	KCNK6	LOC103025301
CCDC25	KHDC4	LOC103025404
CCSER1	KIAA1107	LOC103025426
CDR2L	KIAA1109	LOC103025756
CELF2	KIAA1522	LOC103025817
CEP120	KIAA1671	LOC103025898
CLEC16A	KLHL18	LOC103025952
CLHC1	LIPE	LOC103026087
CLUL1	LNPK	LOC103026151
CMPK1	LOC103021348	LOC103026158
CMPK2	LOC103021388	LOC103026225
COL25A1	LOC103021542	LOC103026350
CSNK2B	LOC103021598	LOC103026594
DDX11	LOC103021919	LOC103026618
DESI2	LOC103021937	LOC103026744
DOK6	LOC103022185	LOC103026850
DSTYK	LOC103022226	LOC103026918
DUSP27	LOC103022502	LOC103026961
EAF2	LOC103022787	LOC103027025
EML1	LOC103022832	LOC103027070
EPHX2	LOC103022898	LOC103027080
ESCO2	LOC103023116	LOC103027108
ETV1	LOC103023166	LOC103027186
EYS	LOC103023221	LOC103027443
FAM13A	LOC103023504	LOC103027450
FAM241A	LOC103023618	LOC103027475
FAM47C	LOC103023655	LOC103027764
FST	LOC103024156	LOC103027893
GADD45A	LOC103024232	LOC103028100
GDPD1	LOC103024407	LOC103028137
GFRA4	LOC103024577	LOC103028327
GLRX	LOC103024654	LOC103028388

LOC103028431	LOC103033816	LOC103041108
LOC103028477	LOC103033881	LOC103041268
LOC103028601	LOC103034116	LOC103041345
LOC103028648	LOC103034997	LOC103041628
LOC103029016	LOC103035038	LOC103041780
LOC103029343	LOC103035617	LOC103041826
LOC103029370	LOC103035809	LOC103041846
LOC103029526	LOC103035951	LOC103041934
LOC103029568	LOC103036004	LOC103042111
LOC103029852	LOC103036092	LOC103042143
LOC103029876	LOC103036502	LOC103042371
LOC103029921	LOC103037404	LOC103042420
LOC103029962	LOC103037517	LOC103042637
LOC103030246	LOC103037523	LOC103042652
LOC103030391	LOC103037579	LOC103043055
LOC103030841	LOC103037674	LOC103043079
LOC103030872	LOC103037998	LOC103043115
LOC103030885	LOC103038058	LOC103043156
LOC103030943	LOC103038316	LOC103043202
LOC103031014	LOC103038317	LOC103043269
LOC103031077	LOC103038408	LOC103043276
LOC103031216	LOC103038664	LOC103043301
LOC103031263	LOC103038914	LOC103043437
LOC103031336	LOC103039006	LOC103043467
LOC103031517	LOC103039085	LOC103043630
LOC103031755	LOC103039110	LOC103043783
LOC103032028	LOC103039298	LOC103043808
LOC103032103	LOC103039619	LOC103043895
LOC103032125	LOC103039690	LOC103044020
LOC103032234	LOC103039742	LOC103044023
LOC103032294	LOC103039911	LOC103044355
LOC103032413	LOC103039934	LOC103044375
LOC103032734	LOC103040030	LOC103044430
LOC103032969	LOC103040205	LOC103044527
LOC103032982	LOC103040475	LOC103044598
LOC103033078	LOC103040476	LOC103044780
LOC103033392	LOC103040561	LOC103044908
LOC103033459	LOC103040571	LOC103045023
LOC103033531	LOC103040666	LOC103045448
LOC103033672	LOC103040995	LOC103045627

LOC103045744	LOC111191359	LOC111194017
LOC103045760	LOC111191382	LOC111194034
LOC103046056	LOC111191388	LOC111194087
LOC103046257	LOC111191391	LOC111194215
LOC103046360	LOC111191409	LOC111194235
LOC103046641	LOC111191623	LOC111194306
LOC103046671	LOC111191628	LOC111194328
LOC103046842	LOC111191630	LOC111194329
LOC103046859	LOC111191665	LOC111194330
LOC103047026	LOC111191730	LOC111194614
LOC103047048	LOC111191763	LOC111194615
LOC103047362	LOC111191800	LOC111194726
LOC103047608	LOC111191862	LOC111194743
LOC103047809	LOC111191864	LOC111194888
LOC107197051	LOC111191888	LOC111194889
LOC107197092	LOC111191976	LOC111194890
LOC107197101	LOC111192051	LOC111194989
LOC107197227	LOC111192062	LOC111195020
LOC107197329	LOC111192378	LOC111195097
LOC107197471	LOC111192397	LOC111195104
LOC107197656	LOC111192512	LOC111195105
LOC111188314	LOC111192539	LOC111195171
LOC111188773	LOC111192579	LOC111195182
LOC111188904	LOC111192626	LOC111195396
LOC111189542	LOC111192630	LOC111195397
LOC111189593	LOC111192701	LOC111195398
LOC111189599	LOC111193054	LOC111195488
LOC111189672	LOC111193082	LOC111195507
LOC111189673	LOC111193087	LOC111195553
LOC111189674	LOC111193225	LOC111195738
LOC111189675	LOC111193234	LOC111195938
LOC111190101	LOC111193241	LOC111196042
LOC111190102	LOC111193463	LOC111196132
LOC111190104	LOC111193492	LOC111196234
LOC111190801	LOC111193541	LOC111196391
LOC111190802	LOC111193710	LOC111196682
LOC111191036	LOC111193719	LOC111196811
LOC111191144	LOC111193730	LOC111196872
LOC111191189	LOC111193837	LOC111196873
LOC111191268	LOC111193890	LOC111197088

LOC111197090
LOC111197165
LOC111197441
LYST
MALRD1
MAP3K15
MAP3K7CL
MAPKAPK3
MFSD9
MICALL1
MTMR6
NAALADL1
NDUFA4L2
NDUFAF6
OCA2
OIT3
PAPPA
PCDH9
PER3
PGGHG
PHTF2
PIGP
PLEKHA8
PPP2R5A
PRPS1
RASA3
RPUSD2
RSRC2
SCEL
SDHAF2
SEMA6D
SFMBT2
SGCD
SGF29
SGMS1
SLC2A9
SMS
SPHKAP
TBC1D9
TDRKH
TLK2
TMEM71
TMEM8B
TRDN
TRNAE-CUC
TRNAI-AAU
TTC12
TTC21B
TTC6
URB2
UTP18
UXS1
VILL
VPS13A
VSIG10L2
XPA
XPO7
YPEL1
ZNF385D
ZNF536
ZNFX1

Supplementary Table 8. List of Primers.

**Primer Name**	**Sequence 5'-3'**
Complementation-forward primer	CCCAAAGCAGAGTGTTTGGTA
Complementation-reverse primer	TTTCCAAAGATCACATATCTTGACA
CRISPR-Oligo A	TAATACGACTCACTATAGGTGTAGCTGAAACGTGGTGAGTTTTAGAGCTAGAAATAGC
CRISPR-Oligo B	GATCCGCACCGACTCGGTGCCACTTTTTCAAGTTGATAACGGACTAGCCTTATTTTAACTTGCTATTTCTAGCTCTAAAAC
CRISPant-forward primer	AGCCCGGACCGTAAGAAG
CRISPant-reverse primer	GCTGTAAACGTCGGGGTAGT
WMISH-forward primer	ATGCGTCTTGTCGGGGCTCAGTATCAG
WMISH-reverse primer	GTTCTGATGTTTACCATGTCTTCCC
