# Supplementary material for: A chromosome-level genome of Astyanax mexicanus surface fish for comparing population-specific genetic differences contributing to trait evolution
Source: Nat Commun. 2021 Mar 4;12:1447. doi: 10.1038/s41467-021-21733-z (PMC7933363; doi:10.1038/s41467-021-21733-z)
Supplement: Supplementary file 3 — Descriptions of Additional Supplementary Files [file 41467_2021_21733_MOESM3_ESM.pdf]

Descriptions of Additional Supplementary Files

Supplementary Data 1

Description: Compiled QTL maps from seven studies referenced to the map from O'Quin et al. 2012. Markers were placed on the v2 Astyanaz Surfacefish Genome assembly using BLASTn. All studies except Kowalko et al. 2013 Current Biology (Marker ID beginning with JK) shared a common set of markers. Genetic map position in cM could therefore not be inferred for the Kowalko et al. 2013 Current Biology markers relative to the other markers. By compiling maps visually across studies, location of some markers could be inferred (e.g. denoted as "placement in Protas 2007").
