# Supplementary material for: A chromosome-level genome of Astyanax mexicanus surface fish for comparing population-specific genetic differences contributing to trait evolution
Source: Nat Commun. 2021 Mar 4;12:1447. doi: 10.1038/s41467-021-21733-z (PMC7933363; doi:10.1038/s41467-021-21733-z)
Supplement: Supplementary file 5 — Reporting Summary [file 41467_2021_21733_MOESM5_ESM.pdf]

Corresponding author(s): Nicolas Rohner, Wesley Warren

Last updated by author(s): Jan 14, 2021

Reporting Summary

Nature Research wishes to improve the reproducibility of the work that we publish. This form provides structure for consistency and transparency in reporting. For further information on Nature Research policies, see our Editorial Policies and the Editorial Policy Checklist.

Statistics

For all statistical analyses, confirm that the following items are present in the figure legend, table legend, main text, or Methods section.

n/a	Confirmed	
☐	☒	The exact sample size (*n*) for each experimental group/condition, given as a discrete number and unit of measurement
☐	☒	A statement on whether measurements were taken from distinct samples or whether the same sample was measured repeatedly
☐	☒	The statistical test(s) used AND whether they are one- or two-sided<br>*Only common tests should be described solely by name; describe more complex techniques in the Methods section.*
☒	☐	A description of all covariates tested
☐	☒	A description of any assumptions or corrections, such as tests of normality and adjustment for multiple comparisons
☐	☒	A full description of the statistical parameters including central tendency (e.g. means) or other basic estimates (e.g. regression coefficient) AND variation (e.g. standard deviation) or associated estimates of uncertainty (e.g. confidence intervals)
☐	☒	For null hypothesis testing, the test statistic (e.g. F, t, r) with confidence intervals, effect sizes, degrees of freedom and P value noted<br>*Give P values as exact values whenever suitable.*
☒	☐	For Bayesian analysis, information on the choice of priors and Markov chain Monte Carlo settings
☒	☐	For hierarchical and complex designs, identification of the appropriate level for tests and full reporting of outcomes
☒	☐	Estimates of effect sizes (e.g. Cohen's *d*, Pearson's *r*), indicating how they were calculated

Our web collection on statistics for biologists contains articles on many of the points above.

Software and code

Policy information about availability of computer code

Data collection	no software was used
Data analysis	bwa mem v0.7.17<br>SAMTools v1.9<br>manta v1.6.0<br>Smoove v0.2.3<br>Nextflow v20.07.1.5412<br>R (v.3.5.3)<br>R/qtl (v.1.44-9)<br>CLC sequence viewer 7 (version 7.7.1)<br>VMD 1.9.3<br>Swiss Model (https://swissmodel.expasy.org/)<br>DIM-Pred (https://www.iitm.ac.in/bioinfo/DIM_Pred/)<br>iStable (http://predictor.nchu.edu.tw/istable/)<br>MUpro (http://mupro.proteomics.ics.uci.edu/)<br>ImageJ (version 1.53g)<br>Graphpad Prism (version 9.0.0)

For manuscripts utilizing custom algorithms or software that are central to the research but not yet described in published literature, software must be made available to editors and reviewers. We strongly encourage code deposition in a community repository (e.g. GitHub). See the Nature Research guidelines for submitting code & software for further information.

Data

Policy information about availability of data

All manuscripts must include a data availability statement. This statement should provide the following information, where applicable:

- Accession codes, unique identifiers, or web links for publicly available datasets
- A list of figures that have associated raw data
- A description of any restrictions on data availability

The raw sequencing and final assembly data generated in this study have been deposited in the NCBI Bioproject database under accession code PRJNA89115 [https://www.ncbi.nlm.nih.gov/bioproject/PRJNA89115/]. The resequencing data used in this study are available in the NCBI BioProject database under accession code PRJNA260715 [https://www.ncbi.nlm.nih.gov/bioproject/PRJNA260715]. All code used to generate the data in this paper is available on github under 10.5281/zenodo.4433170. Original data underlying this manuscript can be accessed from the Stowers Original Data Repository at http://www.stowers.org/research/publications/libpb-1528, found within the article or made available by the authors on request.

Field-specific reporting

Please select the one below that is the best fit for your research. If you are not sure, read the appropriate sections before making your selection.

[x] Life sciences   [ ] Behavioural & social sciences   [ ] Ecological, evolutionary & environmental sciences

For a reference copy of the document with all sections, see nature.com/documents/nr-reporting-summary-flat.pdf

Life sciences study design

All studies must disclose on these points even when the disclosure is negative.

Sample size	For the QTL studies, sample sizes were based on previous studies and general recommendations on reliably performing QTL studies (PMID: 32488995; 30462998; 30042884; 28182695; 27503356; 24035545; 23437360; 17306543). For complementation studies and CRISPR phenotype studies we quantified from all available animals for number of albino oca2 fish and surviving rx3 fish
Data exclusions	none
Replication	Due to the nature of the experiments (QTL, complementation tests, CRISPR knockout studies) replication studies are not commonly used due to the amount of work and the nature of the experiment. A QTL analysis or a complementation experiment is a genetic experiment that provides a definite answer based on the genetics and usually does not require replication. Same is in principle true for CRISPR Knockout studies, usually it is convincing enough to show the correlation of genetic lesion and phenotype. However, to exclude off target effects we repeated the experiment twice, once in adults once in juveniles. For the in situ experiments, these were performed ten times by four different investigators.
Randomization	Randomization is difficult when members of different populations can be visually distinguished, e.g surface fish and cavefish. However, there was also no need for randomization for the types of experiments presented in this study (see comments on Replication and Blinding).
Blinding	The phenotyping occured before the genotyping, as such all experiments were performed blinded.

Reporting for specific materials, systems and methods

We require information from authors about some types of materials, experimental systems and methods used in many studies. Here, indicate whether each material, system or method listed is relevant to your study. If you are not sure if a list item applies to your research, read the appropriate section before selecting a response.

Materials & experimental systems

n/a	Involved in the study	
x		Antibodies
x		Eukaryotic cell lines
x		Palaeontology and archaeology
	x	Animals and other organisms
x		Human research participants
x		Clinical data
x		Dual use research of concern

Methods

n/a	Involved in the study	
x		ChIP-seq
x		Flow cytometry
x		MRI-based neuroimaging

Animals and other organisms

Policy information about studies involving animals; ARRIVE guidelines recommended for reporting animal research

Laboratory animals	Surface population and cave population of Astyanax mexicanus. An adult female was used for the genome sequence. Rest of the animals were not sexed (either too young or not necessary for the phenotypes under study: the presence of eyes or pigmentation is independent of the sex of the animal).
Wild animals	none
Field-collected samples	none
Ethics oversight	The experiments were approved by the institutional animal care and use committees of the institutes that were part of this study.

Note that full information on the approval of the study protocol must also be provided in the manuscript.
